# COVID-19 Patients with Early Gastrointestinal Symptoms Show Persistent Deficits in Specific Attention Subdomains

**DOI:** 10.3390/jcm12051931

**Published:** 2023-03-01

**Authors:** Juliana Schmidt, Maria Cruz, Julio Tolentino, Aureo Carmo, Maria Paes, Glenda de Lacerda, Ana Gjorup, Sergio Schmidt

**Affiliations:** Postgraduate Neurology Department of Neurology, Federal University of the State of Rio de Janeiro, Rio de Janeiro 202709001, Brazil

**Keywords:** attention, cognition, COVID-19, continuous visual attention test, gastrointestinal symptoms

## Abstract

Previous studies have shown that COVID-19 inpatients exhibited significant attentional deficits on the day of discharge. However, the presence of gastrointestinal symptoms (GIS) has not been evaluated. Here, we aimed to verify: (1) whether COVID-19 patients with GIS exhibited specific attention deficits; (2) which attention subdomain deficits discriminated patients with GIS and without gastrointestinal symptoms (NGIS) from healthy controls. On admission, the presence of GIS was recorded. Seventy-four physically functional COVID-19 inpatients at discharge and sixty-eight controls underwent a Go/No-go computerized visual attentional test (CVAT). A Multivariate Analysis of Covariance (MANCOVA) was performed to examine group differences in attentional performance. To discriminate which attention subdomain deficits discriminated GIS and NGIS COVID-19 patients from healthy controls, a discriminant analysis was applied using the CVAT variables. The MANCOVA showed a significant overall effect of COVID-19 with GIS on attention performance. The discriminant analysis indicated that the GIS group could be differentiated from the controls by variability of reaction time and omissions errors. The NGIS group could be differentiated from controls by reaction time. Late attention deficits in COVID-19 patients with GIS may reflect a primary problem in the sustained and focused attention subsystems, whereas in NGIS patients the attention problems are related to the intrinsic-alertness subsystem.

## 1. Introduction

The bidirectional connection between the brain and the gut has been a topic of great interest in the last decade [1]. The brain–gut axis includes the central nervous system, the hypothalamic–pituitary–adrenal axis, the enteric innervation, and more recently the intestinal microbiota [2]. Several studies have shown that disturbances in the microbiota-gut–brain axis are involved in the physiopathology of many disorders, including functional gastrointestinal disorders, Alzheimer’s disease, Parkinson’s disease, Attention-deficit/hyperactivity disorder, and COVID-19 [3,4,5,6,7]. In addition, recent evidence suggests an association between gastrointestinal dysfunction and cognitive impairment [8].

COVID-19, a respiratory disease at origin, has emerged as a systemic disease related with several extrapulmonary manifestations, including gastrointestinal symptoms (GIS) [9]. GIS such as diarrhea, nausea, anorexia, and abdominal pain have been described in more than 10% of COVID-19 patients [10,11,12]. A multicenter study with 106 patients infected by COVID-19, including 40% with GIS, described that almost half of the participants presented an acute mucosal injury [12]. Of note, most of the endoscopy findings were found early during hospital admission. SARS-CoV-2 may invade the gastrointestinal tract through angiotensin-converting enzyme 2 (ACE) receptors and Type II transmembrane serine protease, which are present in the brush border and ciliated cells of intestinal enterocytes [13]. As a result, SARS-CoV-2 infection might cause mucosal damage and thus increase mucosal permeability, allowing bacterial translocation and potentially inflammation [13]. Gut microbiota dysbiosis has also been described in COVID-19 patients and may be an indirect result of ACE-2 dysregulation [7]. Thus, ACE-2 regulation is associated with gut microbiota balance, and its disturbance may play a role in the gastrointestinal symptoms reported by COVID-19 patients [7].

It has been hypothesized that after invading the gastrointestinal tract, SARS-CoV-2 may reach the central nervous system through the vagal nerve, vascular, and/or lymphatic systems [10]. Bostanciklioglu proposed that virus-welded gut inflammation may affect cognitive functions via the vagus nerve [10]. Accordingly, cognitive problems have also been described in COVID-19 patients after hospital discharge [14,15,16,17,18,19,20]. The cognitive deficits include executive functions, memory, and attention [14,15,16,17,18,19,20]. Recently, do Carmo et al. (2022) demonstrated significant attentional deficits in post-COVID patients [19].

In COVID-19 patients after hospital discharge, delayed cognitive deficits may operate independently or instead secondary to reductions in lower order domains, such as basic attention [21]. Attention consists of four subdomains, referred to as intrinsic alertness, sustained attention, focused attention, and behavioral inhibition [22]. These subdomains can be assessed with Go/No-go tests, such as the Continuous Visual Attention Test (CVAT) [22,23,24,25]. It is worth mentioning that the CVAT scores are independent of educational level [22,23,24]. Therefore, a short, computerized education-free attention task could provide relevant information on the cognitive outcome of patients with COVID-19 at the discharge day. As cognitive deficits can be attributed to different attentional subdomains, untangling their relative contribution may help clarify the cognitive impairments observed in post-COVID-19 patients with GIS on the admittance day. Despite the influence of the enteric system on cognition and the role played by the attention subdomains on cognitive performance, there is a lack of information on this subject. We hypothesize that the presence of GIS at baseline would be associated with the presence of persistent attention deficits in COVID-19 patients on the day of discharge from the hospital.

The present study aimed to verify whether COVID-19 patients with GIS at baseline exhibit specific attention subdomain deficits on the day of discharge from the hospital. We also verified which attention subdomain deficits discriminated COVID-19 patients with GIS and without gastrointestinal symptoms (NGIS) from the control group.

## 2. Materials and Methods

### 2.1. Participants (Patients and Controls)

COVID-19 patients (positive RT-PCR) for SARS-CoV-2 were recruited from two reference hospitals in Rio de Janeiro, Brazil. Thirty patients were recruited at the University Hospital and assessed by Aureo do Carmo. This subsample is detailed by Do Carmo et al. (2022) [19]. Another 42 patients were recruited at Lagoa Federal Hospital and assessed by Maria Alice Paes. The symptoms at admittance were recorded for all patients. The patients who, at hospital admission, declared to have acute onset of nausea, vomiting, abdominal pain, diarrhea, and constipation were included in the GIS group. The functional status of COVID-19 patients on the day of discharge was assessed by the physician on duty on that day. The patient should be able to eat, walk, and use a toilet without assistance. At the day of discharge, the patients performed a visual attention task (CVAT).

The control group consisted of age- and sex-matched subjects who had not had a previous infection with SARS-CoV-2 before the CVAT assessment. The controls included hospital employees, patients’ relatives, and volunteers who agreed to participate in the study. They all performed the CVAT.

Exclusion criteria for all groups were: age higher than 70 or <18 years; use of antiseizure or psychotropic drugs; reduced kidney or hepatic function; past head trauma or loss of consciousness; alcohol/substance abuse; pre-existing neurologic or psychiatric disorders; non-corrected hearing or visual impairments; and previous cognitive impairment.

We excluded COVID-19 patients who presented delirium, new neurological symptoms, or orotracheal intubation. Criteria for discharge were a minimum oxygen saturation of 94% in ambient air without oxygen supplementation for 24 h.

The participation was voluntary, and the research protocol was approved by the Gaffrée and Guinle University Hospital Ethics Committee (CAA: 30547720.3.0000.0008). The study was performed in accordance with the Helsinki Declaration. Informed written consent was obtained from all the participants.

### 2.2. Attention Task (CVAT) (Figure 1)

Subjects were seated in front of a computer. The distance between the center of the monitor and the eyes was approximately 50 cm. The examiner instructed the subject to press the spacebar on the keyboard as fast as possible each time a specific target was displayed. The test started with instructions and a practice session. There was one block with 90 trials (two figures presented, one each time, target or non-target). The interstimulus time interval was 1 s. Each stimulus was displayed for 250 milliseconds. The test took 1.5 min to complete. The types of measures included omission errors (OE, focused attention), commission errors (CE, response inhibition), average reaction time of correct responses (RT, intrinsic alertness), and variability of correct reaction times (VRT, sustained attention). VRT was estimated by a per-person measure of the standard deviation (SD) of individual RTs for the correct responses. The total number of correct targets was 72. The participants had to reach more than 50% of the total correct hits (minimum number of correct RT measurements per participant = 37). Previous studies have shown that RT and VRT can be reliably measured by tests as short as 52 s with 20 items [26]. This short version of the CVAT has been applied in previous studies, including for individuals with Alzheimer’s disease and mild cognitive impairment, COVID-19 patients, and in healthy subjects [19,23,24,27].

**Figure 1:**
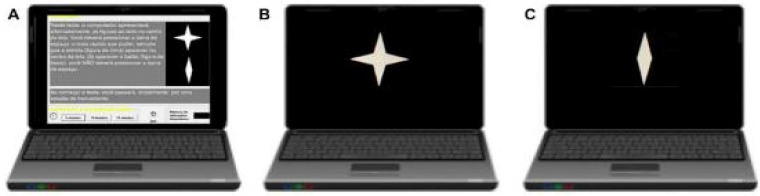
Schematic overview of the set-up of the attentional test (CVAT). (**A**)—The CVAT starts with the following instructions: “In this test, the computer alternately displays the indicated figures in the center of the screen. You must press the spacebar using your dominant hand as fast as you can whenever the star appears in the center of the screen. If the other figure appears, you should not press the space”. (**B**)—The target (star) stays on the screen for 250 milliseconds. (**C**)—The non-target (diamond) remains on the screen for 250 ms. The test has 90 trials and takes 1.5 min to complete. Variables provided by the test: omission errors, commission errors, average Reaction Time of the correct responses (RT), and Intraindividual Variability of Reaction Time (standard deviation of the RTs during the test). The CVAT is open for research and for clinical use (licensed psychologists), upon request to Prof. Sergio L. Schmidt (corresponding author). The test has versions in English, Spanish, and Portuguese. All patients and controls were from Brazil and the Portuguese version was used.

### 2.3. Statistical Analysis

Demographic variables were analyzed using independent sample t-tests for normally distributed continuous variables or chi-square tests for categorical variables. All the following statistical procedures were performed using the dependent variables (OE, CE, RT, and VRT).

Regarding the sample size, we calculated the minimum number of subjects that would be necessary to find clinically significant differences for each CVAT variable. Based on a previous larger sample of healthy subjects, considering α = Type I error = 0.05 and β = Type II error = 0.20 (power= 1 − β = 0.80), we estimated that the minimum number of subjects was 15. It should be mentioned that the CVAT is very reliable in the age range of this investigation.

To assess the effect of COVID-19 on attention subdomains, a Multivariate Analysis of Covariance (MANCOVA) was performed to examine group differences (COVID-19 vs. controls) for the CVAT variables, using age and sex as covariates. In case of a significant overall MANCOVA, post hoc analysis of covariance (ANCOVAs) for each dependent variable was conducted for statistical significance. For the MANCOVA and each of the ANCOVAs, η^2^ (Eta-squared) was computed to calculate the effect size of the results. Cohen has suggested that η^2^ = 0.01 should be considered a small effect size, 0.06 a medium effect size, and 0.14 a large effect size [28].

The procedures described above are all based on mean comparisons. Comparing averages can indicate variable between-group CVAT differences. However, it does not indicate which variables effectively discriminate between groups with and without GI from the control group. Thus, a stepwise discriminant analysis was performed using the raw scores of the attention test (OE, CE, RT, and VRT). Initially, the equality of the group means was tested using Wilk’s λ. Then, the assumptions of the discriminant analysis were tested (linearity, normality, multilinearity, equal variances, and multivariate normal distribution). Box’s M tests were performed to test the assumption of homogeneity of the covariance matrices. The Box’s M test was interpreted in conjunction with inspection of the natural log determinants. The discriminant analysis considered the linear combination of the variables that were necessary and sufficient to discriminate the groups. The canonical discriminant function coefficients were calculated to obtain the Discriminant Functions (DFs). Chi-squared tests (χ^2^) were performed to verify if the DFs were better than chance at separating the groups. Wilk’s λ was used to measure how each function separated the cases. With the aid of each DF, the accuracy of the classification was measured.

To exclude the possibility that a participant’s VRT might be related to the average of the individual RTs, we calculated the coefficient of variability (CV = VRT/RT) for each participant [24]. Therefore, MANCOVAs, ANCOVAs, and the DISCRIMINANT ANALYSES were retested using OE, CE, and CV as dependent variables.

Direct comparisons of the effect of COVID-19 on the different variables of the CVAT were done using standardized non-dimensional scores calculated using the means and the standard deviations of the control group for each CVAT variable. This score showed how many standard deviations a particular patient was above or below the control group mean.

## 3. Results

### 3.1. Demographics (Table 1)

After applying the exclusion and inclusion criteria, 68 controls, 18 eligible COVID-19 inpatients for the GIS group, and 56 for the NGIS group were selected. All demographic information can be found in Table 1. For all the participants (*n* = 142), the age ranged from 18 to 69 years (mean = 47.2; standard deviation = 13.4), and 53% were women. The participants included in this study denied the presence of prior gastrointestinal disorder. Length of stay in the hospital ranged from 1–37 days. No significant demographic differences were found among the groups.

**Table 1:** Demographic data.

		COVID-19	GIS	NGIS	Control	All
		(n = 74)	(n = 18)	(n = 56)	(n = 68)	(n = 142)
Female gender (%)		47%	44%	48%	59%	53%
Age (years)	Mean (SD)	50.0 (13.6)	50.6 (12.5)	49.7 (14.0)	44.6 (12.9)	47.2 (13.4)
	Minimum	18	25	18	18	18
	Maximum	69	67	69	68	69
Length of stay (days)	Mean (SD)	12 (7.6)	8.6 (4.3)	11.2 (8.4)	-	-
	Minimum	1	3	1	-	-
	Maximum	37	15	37	-	-

COVID-19 = Patients diagnosed with COVID-19; GIS = Patients with COVID-19 and gastrointestinal symptoms; NGIS = Patients with COVID-19 without gastrointestinal symptoms; Control = People without COVID-19; SD = standard deviation.

### 3.2. Mean Differences Amount the Groups

As the present study included 74 patients and 68 controls, and the sample size for each subgroup was always >15, we concluded that the sample size reached the number required to find clinically reliable conclusions.

After adjusting for the covariates (age and sex), the MANCOVA showed a significant overall effect of COVID-19 on the attention performance in NGIS patients as compared to controls (F = 23.16, df = 4/117, *p* < 0.001, η^2^ = 0.44). The univariate tests showed that COVID-19 in NGIS patients affected OE (F = 28.26, df = 1/120, *p* < 0.001, η^2^ = 0.19), RT (F = 62.37, df = 1 /120, *p* < 0.001, η^2^ = 0.34), and VRT (F = 75.17, df = 1/120, *p* < 0.001, η^2^ = 0.38), but not CE (F = 0.14, df = 1/120, *p* = 0.71, η^2^ = 0.001).

The same was observed in the GIS group. Thus, in patients with GIS at baseline, the MANCOVA showed a significant overall effect of COVID-19 on the attention performance (F = 19.91, df = 4/79, *p* < 0.001, η^2^ = 0.50). The univariate tests in GIS patients showed that COVID-19 affected OE (F = 55.49, df = 1/82, *p* = <0.001, η^2^ = 0.40), RT (F = 39.33, df = 1/82, *p* < 0.001, η^2^ = 0.32), and VRT (F = 46.80, df = 1/82, *p* < 0.001, η^2^ = 0.36), but not CE (F = 0.087, df = 1/82, *p* = 0.77, η^2^ = 0.001).

When VRT and RT were replaced by CV, the effect of COVID-19 on attention performance in NGIS patients remained significant (F = 19.16, df = 3/118, *p* < 0.001, η^2^ = 0.33). The univariate tests confirmed that COVID-19 affected OE (F = 28.26, df = 1/120, *p* < 0.001, η^2^ = 0.19) and CV (F = 49.24, df = 1/120, *p* < 0.001, η^2^ = 0.29). CE was not affected by COVID-19 (F = 0.14, df = 1 /120, *p* = 0.71, η^2^ = 0.001). The same was found in GIS patients. The MANCOVA tests showed that the effect of COVID-19 on attention performance remained significant (F = 22.57, df = 3/80, *p* < 0.001, η^2^ = 0.46). The univariate tests in GIS patients demonstrated that COVID affected OE (F = 55.49, df = 1/82, *p* < 0.001, η^2^ = 0.404) and CV (F = 26.95, df = 1/82, *p* < 0.001, η^2^ = 0.247). In addition, CE was not affected by COVID-19 (F = 0.087, df = 1/82, *p* = 0.77, η^2^ = 0.001).

### 3.3. Discriminant Model (Table 2)

For the NGI group, considering the CVAT variables (OE, CE, RT, VRT), there were two significant discriminant dimensions [χ^2^ (2) = 68.1, *p* < 0.001]. The pooled within-group correlations identified large correlations with the discriminant model: VRT and RT (Table 2). Therefore, VRT and RT were able to discriminate among the groups. The subjects were correctly classified with 77.4 % accuracy. When VRT and RT were replaced by CV, we found only one significant dimension for the NGIS group [χ^2^ (1) = 42.54, *p* < 0.001]. The pooled within-group correlations identified that CV alone was capable of discriminating the control group from the NGI group, with 73.4% accuracy.

**Table 2:** Pooled within-group correlations between discriminating variables and standardized canonical discriminant functions.

Group without Gastrointestinal Symptoms vs. Control Group	Loadings
Variability of reaction time *	0.91
Reaction Time *	0.86
Omission errors (NI)	0.35
Commission errors (NI)	0.37
Group with Gastrointestinal Symptoms vs. Control Group	Loadings
Variability of reaction time *	0.81
Reaction Time (NI)	0.52
Omission errors *	0.89
Commission errors (NI)	0.23

* = Included in the model; NI = Not Included.

**Figure 2:**
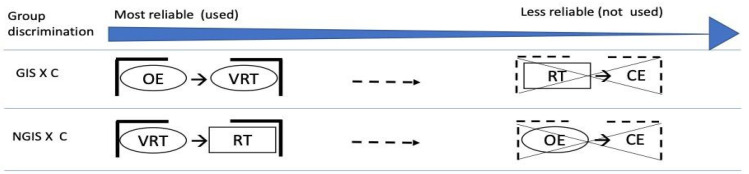
Discriminant analysis. GIS = gastrointestinal symptoms group; NGIS = without gastrointestinal symptoms group; C = control group; 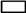 = variable mainly associated with subcortical networks; 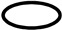 = variable mainly associated with frontoparietal network; OE = omission errors; VRT = variability of the reaction time; RT = reaction time; CE = commission errors. The attention subdomains that are known to be associated with the frontoparietal network are affected in COVID-19 patients who exhibit GIS. In contrast, COVID-19 patients in the NGIS group show a primary deficit in the RT variable, which is known to be associated with subcortical neural structures.

For the GIS group, there were two significant discriminant dimensions [χ^2^ (2) = 53.4, *p* < 0.001]. The pooled within-group correlations identified large correlations with the discriminant model: OE and VRT. Therefore, OE and VRT discriminated GIS patients from controls, and the subjects were correctly classified with 90.7 % accuracy. When VRT and RT were replaced by CV, we still found two significant dimensions for the GIS group [χ^2^ (2) = 50.01, *p* < 0.001]. The pooled within-group correlations identified that CV and OE were capable of discriminating the control group from the GIS group with 90.7% accuracy. The results based on the discriminant analyses are summarized in Figure 2.

### 3.4. Standardized Score

The inspection of the standardized scores based on the control group indicated that in the NGIS group, RT was the most affected variable. In the GIS group, OE and CV were primarily affected. In the GIS group, an increase in CV reflected an increase in VRT independent of RT, as indicated by a non-significant correlation between CV and RT (*p* = 0.17).

## 4. Discussion

The ANCOVAs indicated that RT, VRT, and OE attention subdomains were significantly affected by COVID-19, irrespective of the presence of GIS. However, the discriminant analysis (Figure 1) indicated that the NGIS group could be better differentiated from controls by the effect of COVID-19 on the RT variable. This shows that the primary variable affected in the NGIS group at the baseline is the alertness system (RT). In contrast, the GIS group could be better differentiated from controls by the effect of COVID-19 on the VRT and the OE variables. This indicates that the presence of GIS at the baseline causes a later effect on focused and sustained attention.

### 4.1. How COVID-19 Affects the Gut–Brain Axis

Studies have demonstrated that COVID-19 has the potential to dysregulate the gut–brain axis by promoting systemic inflammation, gut microbiota dysbiosis, and psychological distress [29,30,31,32,33,34]. The virus’s impact on the gut–brain axis may result in neuroinflammation, brain damage, and an increased risk of developing neurodegenerative and neuropsychiatric disorders [29,30,31,32,33,34]. Several authors have proposed that gut dysbiosis may play a major role in the development of neurological disorders in patients infected by SARS-CoV-2 [29,30,31,33,34]. Some of the mechanisms that have been described include impaired production of short-chain fatty acids, increased circulation of lipopolysaccharides, neurotransmitter imbalance, and exacerbated production of proinflammatory cytokines [29,30,31,32,33,34].

### 4.2. The Pronounced RT Increase in Patients without Early Gastrointestinal Symptoms (Figure 2 and Figure 3)

In the present study, the VRT of each participant was estimated by the standard deviation (SD) of the individual’s RTs. Previous studies based on a wide range of different RT tasks have described a linear relation between RT mean and SD [35,36]. Our result in the NGIS group suggests that the general increase in VRT reflects a general slowing of RTs. This is corroborated by our results showing that RT was significantly related to VRT and CV. RT is supposed to be linked to brainstem arousal systems and the anterior cingulate gyrus to maintain alertness [37,38]. Accordingly, Ayalon et al. suggested that a longer average RT is associated with a lower arousal index [39]. Therefore, the greater RT exhibited by COVID-19 patients in the NGIS group may reflect a direct effect of the disease on these brainstem circuits necessary for alertness.

**Figure 3:**
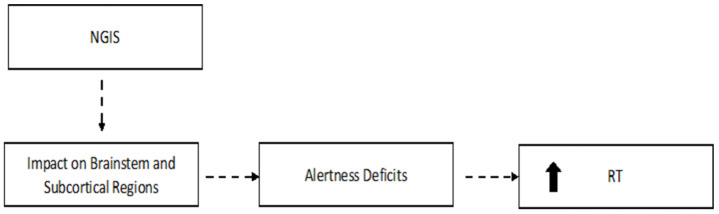
Hypothetical explanation for the attention deficits in the NGIS group. NGIS: COVID-19 patients without gastrointestinal symptoms; RT: reaction time. The arrow indicates an increase in the RT variable.

Young suggested that SARS-CoV-2 may damage the brainstem through viral invasion, inflammation, and vascular activation [40]. As a result, the virus may disrupt neurotransmitter systems in the brain, triggering neurological and cognitive manifestations. It has been proposed that cognitive dysfunction in COVID-19 patients after hospital discharge may be a result of systemic inflammation [41]. In this regard, Zhou et al., in a study including 29 recovered COVID-19 individuals, demonstrated that RT was positively correlated with C-reactive protein, suggesting a general underlying inflammatory process [41]. Indeed, a growing body of evidence suggests that viral infections can cause chronic inflammation, triggering long-lasting cognitive manifestations after the infection period [42]. However, most of the studies including attention assessments were performed with non-respiratory viruses, such as HIV and Hepatitis C [43,44,45,46]. In one of the few studies including a respiratory virus, Smith et al. described that Influenza B affects attentional performance on a simple task by about 20–40% [47]. Conversely, when the subjects were retested, one month later, their RT did not differ from the control group [47].

We suggest that in the NGIS group, the systemic inflammation might be linked with lung dysbiosis. Indeed, a recent study found that COVID-19 patients present higher levels of dysbiosis in the respiratory microbiota compared to healthy controls [48].

### 4.3. The VRT and OE Increase in Patients with Early Gastrointestinal Symptoms (Figure 2 and Figure 4)

The cerebral underpinnings of VRT have been studied less extensively than those of RT itself [49]. Previous studies using a Go/No-Go task in healthy subjects showed that increased VRT was related to an increased response in a network comprising the frontal and parietal regions [50,51].

**Figure 4:**
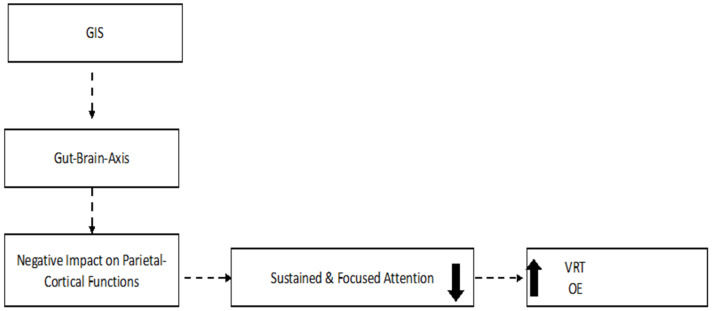
Hypothetical explanation for the attention deficits in the GIS group. GIS = COVID-19 patients with gastrointestinal symptoms; VRT = variability of reaction time; OE = omission errors. The arrows indicate an increase in the VRT and OE variables.

It should be mentioned that GIS may also be a result of dysfunction of the nucleus tractus solitarius and dorsal motor nucleus of the vagus due to its afferent and efferent neuronal projections with the gastrointestinal tract [40]. In addition, previous investigators have suggested that systemic cytokine release may lead to cortical dysfunction, resulting in cerebral hypometabolism and cognitive impartment [52]. Hosp et al. (2021) performed PET scans on average 1 month after symptom onset and observed predominant parietal hypometabolism in two-thirds of 15 COVID-19 patients [52]. Unfortunately, the presence of GIS was not recorded in the study of Hosp et al. (2021).

The higher VRT exhibited by GIS patients might be caused by deficits associated with the sustained attention subdomain, because the VRT variable reflects the stability of response times as the test progressed. The number of omission errors (OEs) was also found to be increased in GIS patients, which indicates a further deficit in the focused-attention subdomain. Simultaneous deficits in OE and VRT might reflect lapses in attention during slow RTs [35]. In the present study, the discriminant analysis gives support for the hypothesis that the results on VRT and OE are explained by the VRT variable in the GIS group. As VRT and OE are related to lapses in attention, we concluded that COVID-19 patients with GIS exhibited sustained attention problems secondary to a primary deficit associated with VRT [25].

### 4.4. The Disrupted Microbiome–Gut–Brain Axis: Early Effect on Vagus Nerve and Later on the Parietal Cortex (Figure 4)

An alternative hypothesis is related to gut dysbiosis and disrupted microbiota–gut–brain axis. Current evidence suggests that microbiota abnormalities are present even after virus clearance and that gut microbiota of COVID-19 patients mirrors disease severity [53]. In addition, in non-COVID-19 patients, the presence of GIS is reflective of gut dysbiosis and disrupted microbiota–gut–brain axis [3]. Thus, we speculate that patients with GIS might present a gut dysbiosis signature that probably impacts the brain attention circuits via the vagus nerve and later the parietal cortex. However, no study has explored the possible association between gut microbiota and cognition dysfunction in post-COVID-19 patients with GIS. Therefore, further investigations are necessary to fully address these hypotheses. Recently, Kohn et al. provided evidence for associative patterns between the gut microbiota and brain network connectivity. They found that an abundance of *Bifidobacterium* was associated with frontoparietal attention networks in healthy subjects [54].

In addition to the brain, gut dysbiosis also affects the lungs [55]. Since SARS-CoV-2 potentially disrupts the gut–brain–lung axis, further studies should investigate if lung dysbiosis plays a role in the cognitive problems observed in COVID-19 patients with GIS at baseline or after infection.

### 4.5. Limitations and Strengths

This article has some limitations. Due to the small sample size, we did not reach statistical power to analyze the influence of other cofactors such as fatigue status, medication use, and comorbidities. In addition, the lack of microbiota and immunological analyses limit the interpretation of the current data. Finally, the study design (cross-sectional) poses a further limitation to the data interpretation. An important strength of this study is the use of an education- and culture-free test.

## 5. Conclusions

In conclusion, the present findings suggest that cognitive problems, commonly observed in COVID-19 patients with GI symptoms, may reflect a primary problem in the sustained and focused attention subsystems. The adequate assessment of the different attention subdomains affected by early GIS would allow a better comprehension of later cognitive consequences of this condition.

## Data Availability

The datasets generated during and/or analyzed during the current study are available from the corresponding author on reasonable request.
